# Anticancer Effects of Amlodipine Alone or in Combination With Gefitinib in Non-Small Cell Lung Cancer

**DOI:** 10.3389/fphar.2022.902305

**Published:** 2022-06-01

**Authors:** Bingjie Fu, Xiaojing Dou, Miao Zou, Hao Lu, Kaixuan Wang, Qingxia Liu, Yao Liu, Wei Wang, Meihua Jin, Dexin Kong

**Affiliations:** ^1^ Tianjin Key Laboratory of Technologies Enabling Development of Clinical Therapeutics and Diagnostics, School of Pharmacy, Key Laboratory of Immune Microenvironment and Diseases (Ministry of Education), Tianjin Medical University, Tianjin, China; ^2^ Department of Otorhinolaryngology Head and Neck Surgery, Key Laboratory of Auditory Speech and Balance Medicine, Institute of Otolaryngology of Tianjin, Tianjin First Central Hospital, Tianjin, China

**Keywords:** amlodipine, gefitinib, lung cancer, synergistic anticancer effect, cell cycle arrest

## Abstract

Amlodipine is a Ca^2+^ channel blocker commonly used to cardiovascular diseases such as hypertension and angina; however, its anticancer effects in lung cancer A549 cells remain unknown. In the present study, we explored the antitumor effects and molecular mechanisms underlying the action of amlodipine in non-small cell lung cancer (NSCLC) A549 cells *in vitro* and *in vivo*. We observed that amlodipine suppressed the proliferation of A549 lung cancer cells by arresting the tumor cell cycle. Mechanistically, our results revealed that amlodipine could attenuate the phosphoinositide 3 kinase (PI3K)/Akt and Raf/MEK/extracellular signal-regulated kinase (ERK) pathways through epidermal growth factor receptor (EGFR) and modulated cell cycle-related proteins such as cyclin D1, p-Rb, p27, and p21. Subsequently, amlodipine combined with gefitinib could synergistically inhibit cell proliferation by arresting the cell cycle. Moreover, amlodipine combined with gefitinib effectively attenuated the growth of A549 lung cancer xenografts when compared with monotherapy, affording an excellent therapeutic effect. Collectively, our results indicate that amlodipine alone or combined with the novel anticancer drug gefitinib might be a potential therapeutic strategy for NSCLC patients with wild-type EGFR.

## Introduction

Calcium channel blockers (CCBs) inhibit calcium flux through voltage-gated calcium channels (Wang et al., 2021) and are widely used to treat cardiovascular diseases such as angina, hypertension, and supraventricular arrhythmias (Sica, 2006). CCBs are classified as dihydropyridines or non-dihydropyridines. Dihydropyridines include nifedipine, nicardipine, felodipine, and amlodipine, while non-dihydropyridines include diltiazem and verapamil (Eisenberg et al., 2004). CCBs, such as diltiazem and verapamil, were shown to decrease tumor growth in a xenograft mouse meningioma tumor model (Jensen and Wurster, 2001), and verapamil could reversibly decrease human melanoma cell invasion and metastasis (Yohem et al., 1991). In addition, dihydropyridine derivatives, such as amlodipine, nicardipine, and nimodipine, reportedly suppressed the growth of human epidermoid carcinoma cells, whereas verapamil, diltiazem, and dihydropyridine nifedipine failed to inhibit human epidermoid carcinoma cell growth at the same concentration (Yoshida et al., 2003). However, the anticancer effect of amlodipine against non-small cell lung cancer (NSCLC) A549 cells remains unknown.

Lung cancer is the most frequent and leading cause of cancer-related deaths globally, and NSCLC constitutes approximately 75% of all lung cancers (Cersosimo, 2002; Hirsch et al., 2017). Epidermal growth factor receptor (EGFR) is a transmembrane protein with cytoplasmic kinase activity commonly overexpressed in various human cancers, including NSCLC (da Cunha Santos et al., 2011; Wee and Wang, 2017). EGFR tyrosine kinase inhibitors (TKIs), gefitinib and erlotinib, are targeted therapies, used as first-line treatments for patients with NSCLC harboring EGFR mutations (Shukuya et al., 2011; De Mello et al., 2016). EGFR-TKIs have demonstrated insufficient efficacy in patients with wild-type EGFR (Hirai et al., 2017). However, it has been reported that shikonin could enhance the anticancer efficacy of gefitinib/erlotinib in wild-type EGFR NSCLC cells (Li et al., 2018). And recently, we also reported that proton pump inhibitor, lansoprazole, in combination with gefitinib showed synergistic antitumor effect on NSCLC A549 cells and mouse xenograft models with wild-type EGFR (Zhao et al., 2021). Therefore, potential EGFR-TKI sensitizers and treatment strategies to improve the prognosis of lung cancer patients with wild-type EGFR are essential.

Combination therapy, rather than monotherapy, can afford synergistic clinical benefits. Amlodipine attenuates phosphorylation of EGFR in human epidermoid carcinoma A431 cells *in vitro* and *in vivo* (Yoshida et al., 2010). Therefore, we hypothesized that simultaneous treatment with amlodipine and gefitinib might have synergistic anticancer effects. In the present study, we investigated the anticancer effects and preliminary mechanisms of amlodipine alone or in combination with gefitinib in NSCLC A549 cells with wild-type EGFR. Our study provides a promising new therapeutic agent or combination treatment strategy for patients with NSCLC.

## Materials and Methods

### Cell Culture

A549 cells were obtained from the Cell Resource Center of Peking Union Medical College (Beijing, China). This cell line has been authenticated using STR profiling within the last 3 years. In addition, A549 cells were confirmed to be free of *mycoplasma* contamination by PCR. Briefly, A549 cells were cultured in RPMI 1640 medium containing 10% fetal bovine serum (FBS), 100 U/ml penicillin, and 100 μg/ml streptomycin. The cell cultures were maintained in a humidified atmosphere containing 5% CO_2_ at 37°C.

### Reagents

Amlodipine and gefitinib were purchased from Selleck Chemicals (Houston, TX, United States) and TopScience (Shanghai, China), respectively. Propidium iodide (PI) was obtained from Sigma-Aldrich (St. Louis, MO, United States). The Annexin V/FITC-PI apoptosis detection kit was obtained from BD Biosciences (San Jose, CA, United States of America). RPMI 1640 and FBS were purchased from Biological Industries (Beit Haemek, Israel). The enhanced chemiluminescence (ECL) reagent was purchased from Thermo Fisher Scientific (Waltham, MA, United States). Antibodies against phospho-EGFR, phospho-PDK1 (Ser241), PDK1, phospho-Akt (Ser473), phospho-Akt (Thr308), Akt, phospho-mammalian target of rapamycin complex 1 (mTORC1) (Ser2448), mTOR, phospho-glycogen synthase kinase 3 (GSK-3) β, phospho-p70 S6K (Thr389), p27, cyclin D1, phospho-retinoblastoma protein (Rb), phospho-extracellular signal-regulated kinase (ERK) 1/2, phospho-c-Raf, *β*-actin, and horseradish peroxidase (HRP)-conjugated goat anti-rabbit and horse anti-mouse secondary antibodies were purchased from Cell Signaling Technology Inc. (Danvers, MA, United States). Antibody specific for p21 was obtained from Santa Cruz Biotechnology Inc. (Dallas, TX, United States).

### Determination of Cell Viability

Cell viability was determined using an 3-(4,5-dimethylthiazol-2-yl)-2,5-diphenyltetrazolium bromide (MTT) assay. Briefly, A549 cells were seeded into 96-well plates at a density of 4×10^3^ cells/well and then treated with amlodipine and/or gefitinib for 48 h, followed by the addition of 20 μl MTT (5 mg/ml) to each well and further incubation for 4 h. Dimethyl sulfoxide (DMSO) was used to dissolve purple crystals after incubation. Optical density was measured at 490 nm using an iMark microplate reader (Bio-Rad, Hercules, CA, United States).

### Flow Cytometric Analysis

Briefly, A549 cells were plated in 6-well plates at a density of 2 × 10^5^ cells/well and treated with amlodipine or gefitinib for 48 h. For cycle analysis, the cells were harvested with trypsin, washed with PBS, resuspended in 75% pre-chilled ethanol, and stored at 4 °C overnight. After 24 h, the fixed cells were collected and stained with PI. Then, 20,000 cells were collected for each sample and analyzed using a BD Accuri C6 flow cytometer (BD Biosciences, San Jose, CA, United States).

To analyze cell apoptosis, Annexin V-FITC/PI double staining was performed as previously reported, with minor modifications (Zhang et al., 2021). The drug-pretreated cells were harvested with trypsin, resuspended in Annexin V-FITC/PI (BD Biosciences, San Jose, CA, United States) mixture, and incubated in the dark for 15 min. Subsequently, cells were analyzed using a BD Accuri C6 flow cytometer (10,000 cells were analyzed for each sample).

Data were quantified with BD Accuri C6 Software.

### Wound-Healing Assay

Briefly, A549 cells were seeded in 24-well plates at a density of 6×10^5^ cells/well and a micropipette tip was used to create a vertical scratch (wound) in the cell monolayer. Subsequently, cells were treated with different concentrations of amlodipine for 48 h. Wound areas were imaged under a microscope.

### Western Blotting

Western blot analysis was performed as previously described (Shao et al., 2021). Briefly, cells were lysed with RIPA lysis buffer, and the protein concentration of each sample was determined using a BCA protein assay kit. Protein extracts were separated by 10% sodium dodecyl sulfate-polyacrylamide gel electrophoresis (SDS-PAGE) and transferred to a PVDF membrane. Membranes were blocked with 5% non-fat milk, incubated with primary antibodies at 4 °C overnight, washed four times with Tris-buffered saline (TBS) containing Tween-20 (TBST), and incubated with the respective HRP-conjugated secondary antibody for 1 h. Protein bands were visualized using ECL reagents on a Bio-Rad ChemiDoc™ XRS + system (Bio-Rad, Hercules, CA, United States).

### Nude Mouse Xenograft Tumor Experiments

All animal experiments were conducted at the Laboratory Animal Center of the Institute of Radiation Medicine, Chinese Academy of Medical Sciences, and were maintained in a pathogen-free environment. The experiments were performed under the Institutional Animal Care and Use Committee guidelines. Six-week-old male BALB/c nude mice were injected subcutaneously (s.c.) with 1 × 10^7^ A549 cells, and then the sufficiently grown tumor tissue was divided into equal pieces, followed by subcutaneous implantation into the mice. Mice were randomly divided into four groups (*n* = 4) when tumors reached 20–30 mm^3^, and then orally administered amlodipine (10 mg/kg) or gefitinib (50 mg/kg) alone or in combination every other day for 15 days. The growth of implanted tumors was monitored every other day, tumor volumes were measured using a caliper, and the tumor volume was calculated using the formula V = 1/2 × Length×Width^2^.

### Immunohistochemistry Analyses

At the end of the experimental period, animals were euthanized, and tumors were excised and fixed in paraformaldehyde for IHC analysis. Tumors were embedded in paraffin, sectioned at 5-μm thickness, and stained with hematoxylin and eosin (H&E) for morphological assessment. The formalin-fixed tissue was heated in an antigen retrieval solution before incubation with the antibody Ki67, and bound antibodies were detected with Bond Polymer (anti-rabbit poly-HRP-IgG) and visualized using diaminobenzidine (DAB) peroxidase substrate. Specimen images were examined using an Olympus BX51 microscope.

### Statistical Analysis

Data values are expressed as the mean ± standard deviation (SD) of triplicate values. One-way ANOVA followed by Tukey’s multiple comparison test was used to determine statistical significance using GraphPad Prism 5 (GraphPad, San Diego, CA, United States). Differences were considered statistically significant at *p* < 0.05.

## Results

### Amlodipine Inhibits the Proliferation of NSCLC A549 Cells *Via* Cell Cycle Arrest at the G0/G1 Phase

**FIGURE 1:**
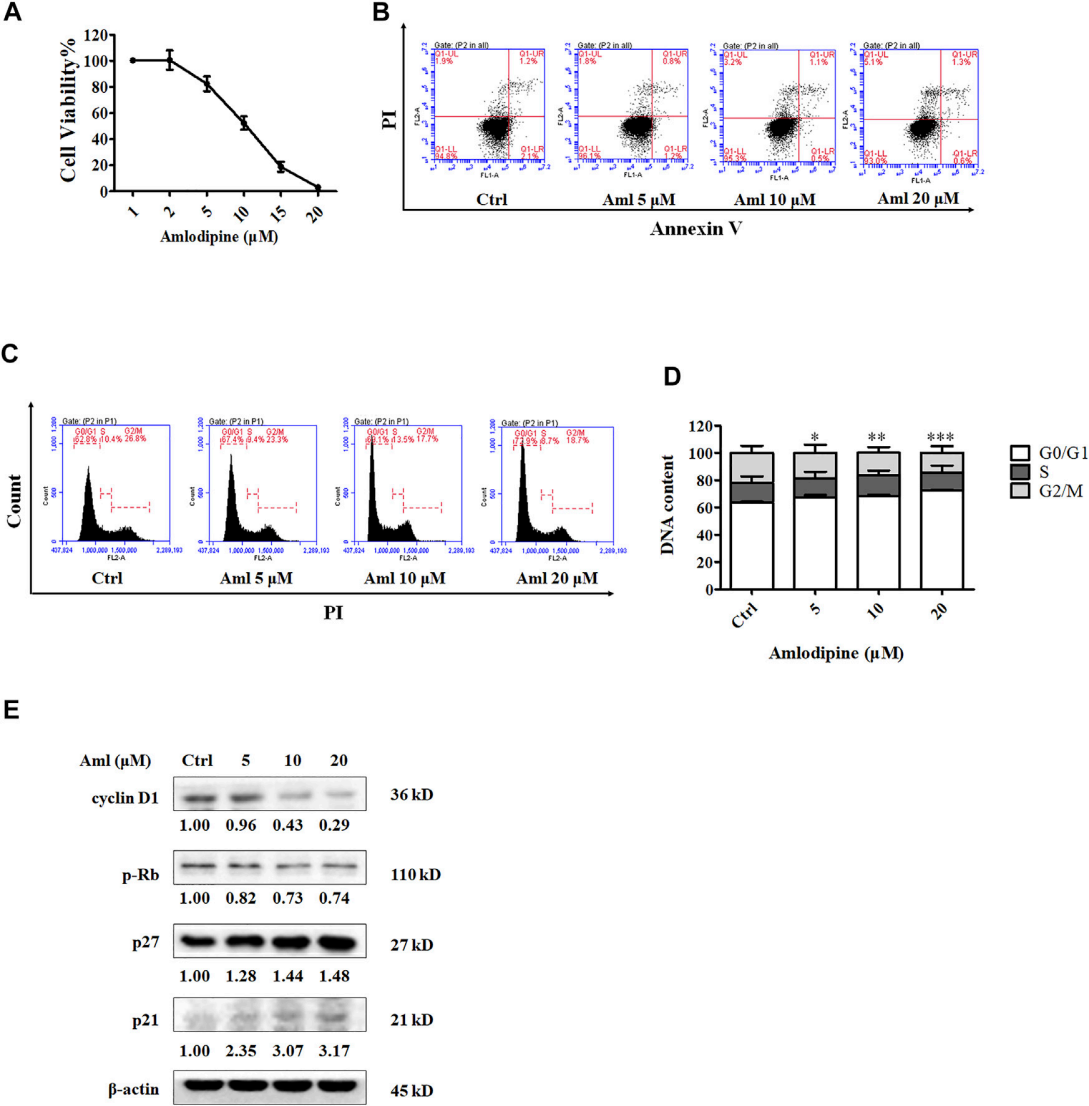
Amlodipine inhibits A549 cell proliferation by blocking the cell cycle G0/G1 phase. **(A)** Incubation of A549 with various concentrations (1, 2, 5, 10, 15, and 20 μM) of amlodipine (Aml) for 48 h. The effects of amlodipine on A549 cell proliferation were determined by MTT assay. **(B)** A549 cells were harvested 48 h after treatment with indicated concentrations of amlodipine, and apoptotic cells were measured by flow cytometry analysis. **(C)** Cells were pre-incubated with different concentrations of amlodipine for 48 h, and the induction of cell cycle arrest was detected by flow cytometry analysis. **(D)** The percentage of total cells at G0/G1, S, and G2/M phases. **(E)** The expressions levels of cell cycle-related proteins were determined by Western blotting after treatment with amlodipine. Data are presented as mean ± SD of three independent experiments. *p < 0.05, **p < 0.01 and ***p < 0.001.

The MTT assay was used to detect the inhibitory effect of amlodipine on A549 cell proliferation. A549 cells were treated with a series of amlodipine concentrations for 48 h. As shown in Figure 1A, amlodipine significantly suppressed the proliferation of A549 cells in a concentration-dependent manner, with an IC_50_ of 9.641 μM. Apoptosis is a common mechanism of cell death. We first determined whether amlodipine induces apoptosis in A549 cells. A flow cytometry assay with double staining using Annexin-V FITC/PI was performed to detect the percentage of apoptotic cells. A549 cells were treated with 5, 10, and 20 μM amlodipine for 48 h. As shown in Figure 1B, incubation with amlodipine showed no significant changes. To explore the effects of amlodipine on the cell cycle, flow cytometry was used to evaluate cell cycle phase distribution in A549 cells following amlodipine treatment for 48 h. As shown in Figures 1C,D, amlodipine-treated cells exhibited significantly enhanced cell cycle arrest at the G0/G1 phase when compared with the non-treated group. To further explore these findings, the levels of cyclin D1, p27, and p21, as well as the phosphorylation of their downstream protein Rb, were determined using Western blotting after treatment with 5, 10, and 20 μM amlodipine for 48 h. Cyclin D-cyclin dependent kinase (CDK) 4/6 complex phosphorylates Rb to facilitate cell cycle progression from G1 to S phase (Sherr, 1994; Shapiro, 2006). As shown in Figure 1E, the expression of cyclin D1 and level of p-Rb were decreased, while the expression levels of p27 and p21 (CDK inhibitors) were increased in a dose-dependent manner, compared with the non-treated group. These findings indicated that amlodipine inhibited A549 cell proliferation by inducing cell cycle arrest but did not significantly affect apoptosis.

### Amlodipine Suppresses A549 Cell Migration

**FIGURE 2:**
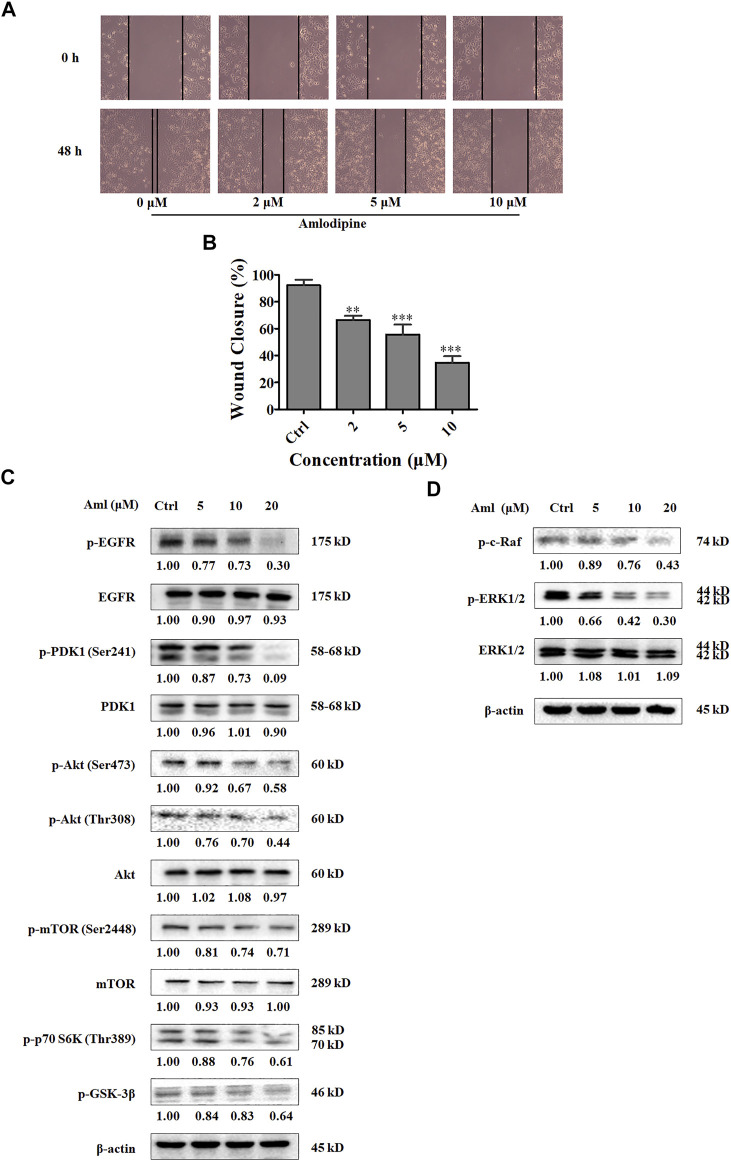
Amlodipine suppresses A549 cells migration and PI3K/Akt and Raf/ERK MAPK pathways. **(A)** The effect of amlodipine on cell migration was detected by performing a wound-healing assay at 48 h. **(B)** Quantification of results in **(A). (C)** The levels of Akt pathway proteins were analyzed by Western blotting after treatment with amlodipine. **(D)** The phosphorylation of Raf and phosphorylation and total ERK1/2 were analyzed by Western blotting after treatment with amlodipine. Data are presented as mean ± SD of three independent experiments. **p < 0.01 and ***p < 0.001.

Metastasis is associated with poor prognosis, and cancer cell migration and invasion are recognized as the initial steps in metastasis. To examine the effects of amlodipine on A549 cell migration, a scratch-wound assay was performed using evenly space 24-well plates covered with A549 cells. Amlodipine was then administered at concentrations of 2, 5, and 10 μM (to avoid interference between cell proliferative activity and migration, we selected a concentration less than the IC_50_), and scratches were examined after 48 h of culture. As shown in Figures 2A,B, cell migration was notably lower in the amlodipine group than in the untreated group, with amlodipine treatment exhibiting a concentration-dependent effect.

### Amlodipine Inhibits PI3Ks/Akt and Raf/MEK/ERK MAPKs Pathways by Inhibiting EGFR Phosphorylation in A549 Cells

The phosphatidylinositol-3 kinase (PI3K) pathway plays an important role in regulating cell migration, proliferation, cell cycle progression, and apoptosis (Martini et al., 2014). Therefore, to explore the molecular mechanism underlying amlodipine-mediated inhibition of A549 cell proliferation and cell cycle arrest, cells were treated with 5, 10, and 20 μM amlodipine for 48 h, and Western blotting was performed to detect PI3Ks pathway proteins. As shown in Figure 2C, amlodipine treatment at 20 μM for 48 h significantly decreased phosphorylation levels of PDK1, Akt (both Ser473 and Thr308), mTOR, p70 S6K, and GSK-3β, without changes in total PDK1, Akt and mTOR levels.

The Ras/Raf/MEK/ERK pathway is the most pivotal signaling cascade among all MAPK signal transduction pathways and regulates the survival, growth, differentiation, and development of tumor cells (Degirmenci et al., 2020; Guo et al., 2020). In addition, to examine the role of MAPK signaling inhibition and the effects of amlodipine on proliferation and migration, A549 cells were pretreated with several amlodipine concentrations for 48 h. As shown in Figure 2D, phosphorylation levels of c-Raf and ERK were reduced in a concentration-dependent manner in cells treated with 10 and 20 μM amlodipine with no variation detected in total ERK, when compared with untreated cells.

EGFR is a receptor tyrosine kinase that plays a critical role in carcinogenesis, including cell proliferation, survival, and differentiation (da Cunha Santos et al., 2011). PI3K and MAPK signaling pathways are involved in EGFR signal transduction. To determine whether amlodipine affects the upstream regulator EGFR, we assessed EGFR phosphorylation after amlodipine treatment using Western blotting. As shown in Figure 2C, amlodipine significantly suppressed the level of p-EGFR when compared with the untreated control group, and the inhibitory effects were particularly significant following treatment with 10 and 20 μM amlodipine with no variation detected in total EGFR.

### Amlodipine in Combination With Gefitinib Synergistically Reduces Cell Proliferation Through Cell Cycle Arrest at G0/G1 by Attenuating PI3K/Akt Signaling Pathway in A549 Cells

**FIGURE 3:**
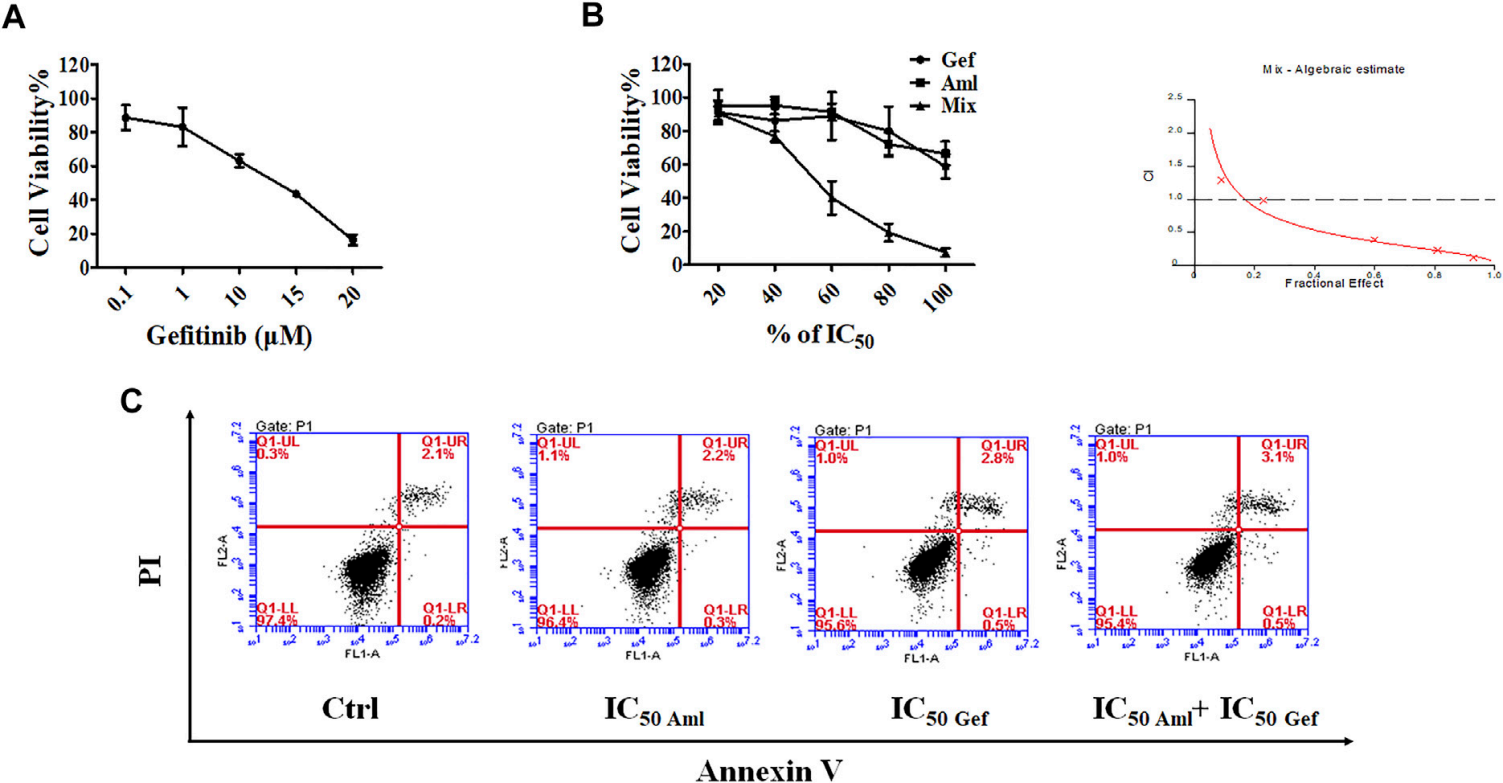
Amlodipine combined with gefitinib led to a synergistic anti-proliferative effect on A549 cells. **(A)** MTT assay was used to detect the inhibitory effect of gefitinib (Gef) on cell proliferation. **(B)** A549 cells were treated with amlodipine, gefitinib, or a combination of these agents for 48 h. A549 cells were treated with amlodipine and gefitinib, with several percentages of IC_50_ value. **(C)** A549 cells were harvested 48 h after treatment with amlodipine, gefitinib, or a combination of these agents, and apoptotic cells were examined by flow cytometry analysis.

Several reports have shown that combination therapies afford synergistic antitumor effects when compared with the efficacy of either agent alone, such as a combination of cisplatin and vinorelbine, which is superior to cisplatin monotherapy in patients with advanced NSCLC (Wozniak et al., 1998). Therefore, gefitinib was selected and combined with amlodipine. First, the IC_50_ of gefitinib at 48 h was detected using the MTT assay, as shown in Figure 3A, notably inhibiting cell proliferation at an IC_50_ of 12.52 μM. To quantify the interaction between amlodipine and gefitinib, the multiple-drug effect evaluation introduced by Chou-Talalay was employed. The MTT assay was performed to measure the inhibitory activities of a series of drug concentration combinations (20, 40, 60, 80, and 100% of IC_50_ values of each drug) (Figure 3B). To detect the efficacy of the two-drug combination, combination index (CI) plots were generated using CalcuSyn software, and CI values were defined as follows; CI < 1, synergism; CI = 1, additive; and CI > 1, antagonism. The CI values for ED_50_, ED_75_, and ED_90_ were 0.43812, 0.26170, and 0.16192, respectively. Therefore, we fixed the ratio of IC_50 amlodipine_:IC_50 gefitinib_ (9.641 μM:12.52 μM) combination to perform a subsequent mechanistic study for examining the synergistic anti-proliferative effect.

**FIGURE 4:**
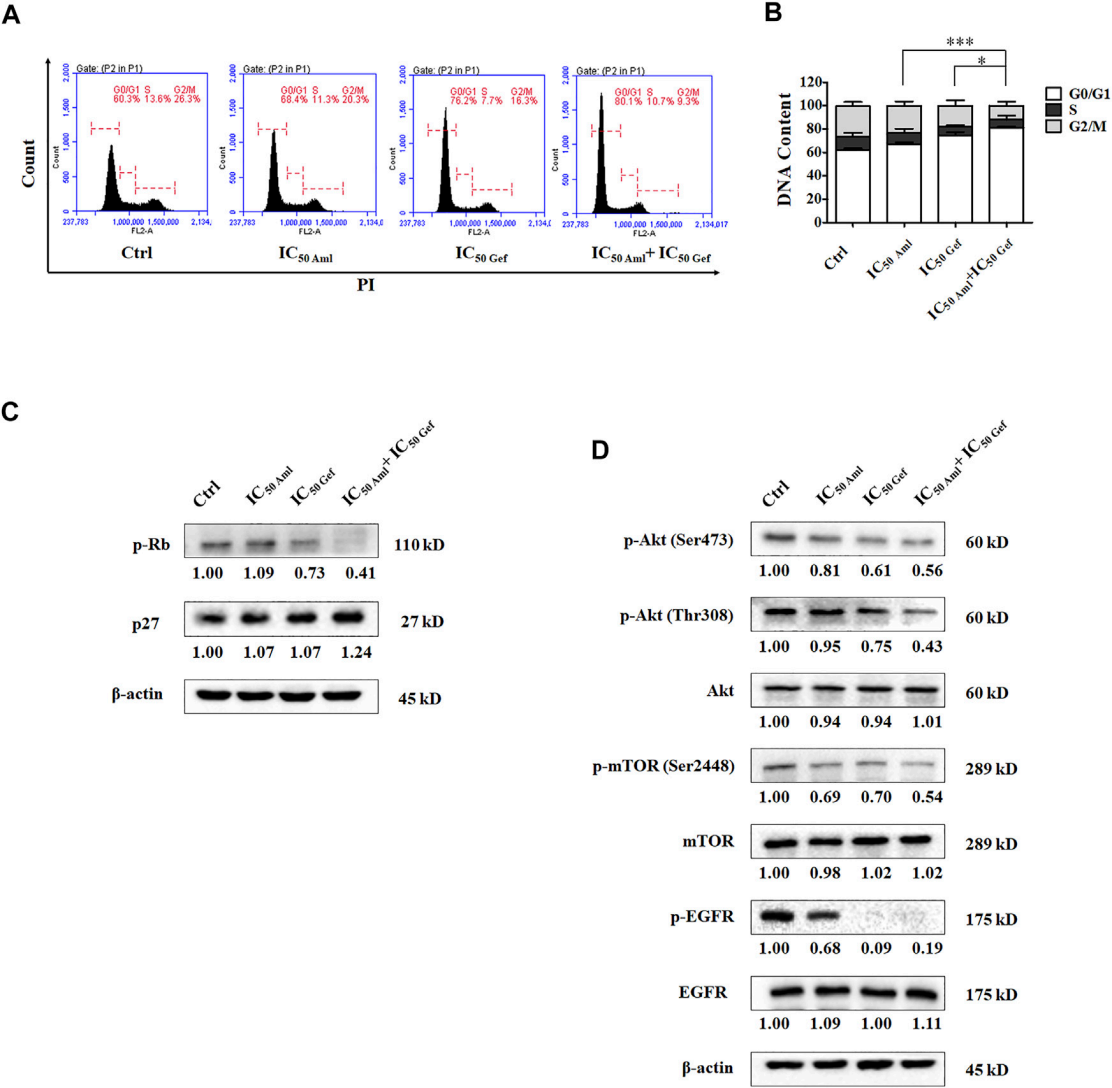
Amlodipine combined with gefitinib induces cycle arrest in A549 cells. **(A)** Amlodipine combined with gefitinib increased the proportion of cells in the G0/G1 phase. **(B)** The percentage of total cells at G0/G1, S, and G2/M phases. **(C)** The levels of p-Rb and p27 were measured by Western blotting after treatment with amlodipine, gefitinib, or a combination of the two agents. **(D)** Phosphorylation and total levels of Akt, mTOR, and EGFR were analyzed by Western blotting after treatment with amlodipine, gefitinib, or a combination of the two agents. Data are presented as mean ± SD of three independent experiments. *p < 0.05 and ***p < 0.001.

We evaluated the effect of amlodipine combined with gefitinib on A549 cell apoptosis using Annexin V-FITC/PI staining. A549 cells were treated with amlodipine and gefitinib, either alone or in combination, for 48 h. The results revealed no significant changes following treatment with either amlodipine or a combination of amlodipine and gefitinib (Figure 3C). We further analyzed the effects of amlodipine in combination with gefitinib on the cell cycle. It has been reported that gefitinib, as well as amlodipine, can block the A549 cell cycle at the G0/G1 phase. Therefore, to clarify the combined anti-proliferation activity detected in the MTT assays, flow cytometry was performed to assess A549 cells treated with the two drugs alone or in combination for 48 h; the percentage of each cell cycle was determined by PI staining. As shown in Figures 4A,B, treatment of A549 cells with amlodipine combined with gefitinib significantly increased the proportion of cells in the G0/G1 phase (80.1%) when compared with amlodipine or gefitinib alone. A mechanistic assessment was performed using Western blotting, and the results indicated that the combined use of amlodipine and gefitinib significantly reduced levels of p-Rb and enhanced p27 when compared with amlodipine or gefitinib alone (Figure 4C).

In addition, the mechanism of action was investigated using Western blotting. Compared with amlodipine or gefitinib treatment alone, amlodipine combined with gefitinib could downregulate PI3K/Akt signaling pathway proteins, including phosphorylation of Akt (Ser473), Akt (Thr308), and mTOR (Figure 4D) with no variation detected in total Akt and mTOR levels. Furthermore, amlodipine combined with gefitinib decreased the phosphorylation of EGFR in A549 cells without any change in total EGFR when compared with the amlodipine alone group (Figure 4D).

### Amlodipine Alone or in Combination With Gefitinib Suppresses A549 Xenograft Tumor Growth

**FIGURE 5:**
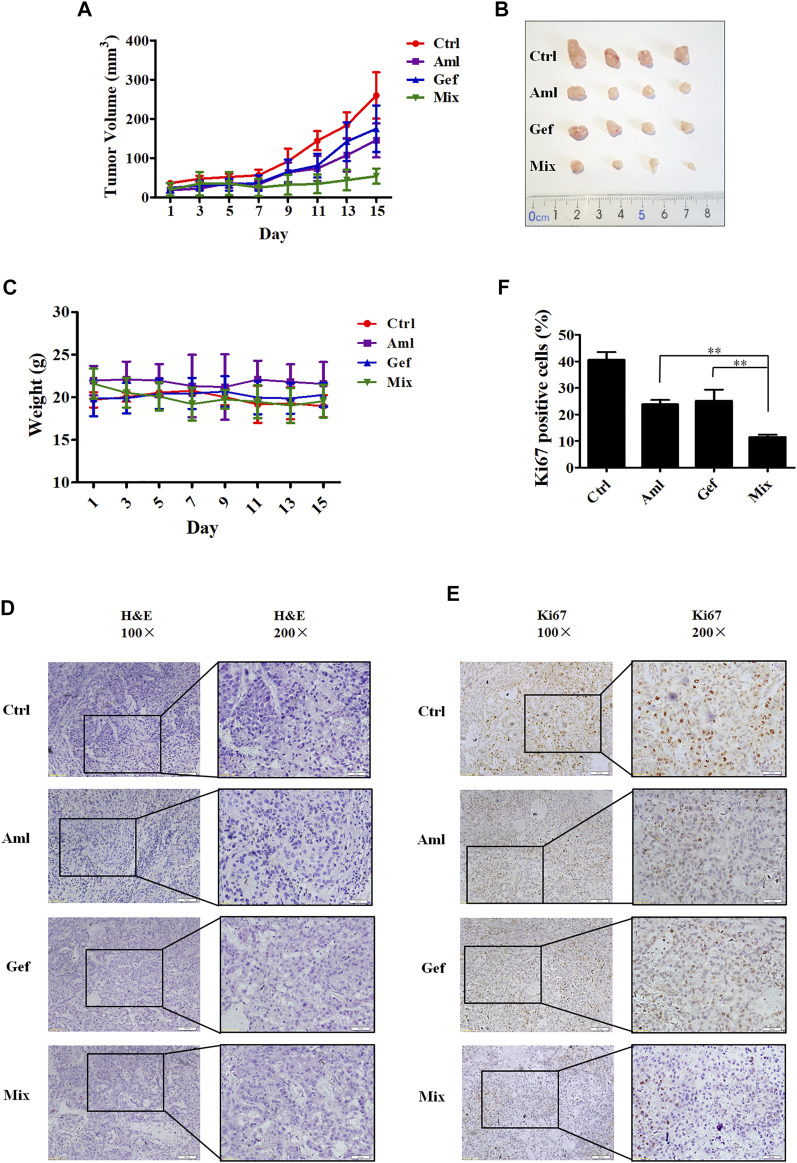
Amlodipine combined with gefitinib inhibits tumor growth in nude mice bearing A549 xenograft. Tumor-bearing xenograft mice were orally administered amlodipine (10 mg/kg every other day) and gefitinib (50 mg/kg every other day), alone or in combination, for 15 days. **(A)** Tumor volumes were measured every alternate day. **(B)** Photographs of the isolated tumors in each group. **(C)** Representative body weights of mice in each group. **(D)** Tumor tissues were stained with H&E. **(E)** Tumor tissues were stained for the proliferation marker Ki67. **(F)** Quantitative analysis of the Ki67 positive cells in tumor sections from mice. Data are presented as mean ± SD obtained from at least 3 mice. **p < 0.01.

Based on the aforementioned results from *in vitro* experiments, we speculated whether amlodipine or its combination with gefitinib could afford antitumor effects *in vivo*. Therefore, we examined the efficacy of amlodipine alone and in combination with gefitinib in an A549 xenograft mouse model. Tumor-bearing xenograft mice were orally administered amlodipine (10 mg/kg) or gefitinib (50 mg/kg), alone or in combination, every other day for 15 days. As shown in Figures 5A,B, amlodipine alone showed significant antitumor activity against A549 tumors, and this dose resulted in no significant weight loss when compared with the non-treated control group (Figure 5C). This result suggests that the test does not induce severe side effects. In addition, treatment with amlodipine combined with gefitinib markedly inhibited the growth of A549 tumors when compared with monotherapy with either agent, with no significant differences in body weight. Furthermore, we compared the histological findings following treatment with amlodipine alone or combined with gefitinib with the untreated group. H&E staining was used to confirm the tissue morphology (Figure 5D). Tumor tissue sections stained with the tumor cell proliferation marker Ki67 are shown in Figure 5E. Amlodipine combined with gefitinib reduced Ki67 positive brown cells when compared with either amlodipine or gefitinib alone (Figure 5F).

## Discussion

Amlodipine, a Ca^2+^ channel blocker, is frequently used to treat cardiovascular diseases, such as hypertension and angina (Sheraz et al., 2016). The efficacy and safety of long-term amlodipine combined with lisinopril were previously reported in patients with hypertension (Arslanagic et al., 2005). Recent studies have shown that amlodipine exhibits antitumor activity; for example Yoshida *et al.* have reported that amlodipine inhibits proliferation of human epidermoid carcinoma A431 cells, induces G1 phase A431 cell accumulation, retards tumor growth, and prolongs the survival of A431 tumor cell-bearing xenograft mice (Yoshida et al., 2003; 2004; 2007). However, the antitumor effects of amlodipine in NSCLC and its underlying antitumor effects have not been reported. In the present study, we found that amlodipine exhibited beneficial antitumor effects against lung A549 cancer cells both *in vitro* and *in vivo*, with no significant side effects. The mechanistic study revealed that amlodipine attenuated cell proliferation through cell cycle arrest at the G0/G1 phase without marked apoptosis induction. This result is consistent with that of Yoshida *et al.*, who reported that the anti-proliferative effect of amlodipine could not be attributed to cytotoxicity (Yoshida et al., 2003), and caused G1 cell cycle arrest and cell growth inhibition in human epidermoid carcinoma cells (Yoshida et al., 2007). In addition, we found that amlodipine arrested the cell cycle by regulating the cell cycle pathway. Western blot analysis revealed that cyclin D1 and p-Rb were significantly downregulated, while p27 and p21 were upregulated in amlodipine-treated A549 cells. There are three known D-type cyclins (D1, D2, and D3) that form active complexes with either CDK4 or CDK6, as well as cyclin E-CDK2 complexes, which phosphorylate Rb to facilitate the G1 to S phase transition (Shapiro, 2006; Qie and Diehl, 2016). CDK activity is negatively modulated by the INK4 and CIP/KIP families, which include p21 and p27 (Donjerkovic and Scott, 2000; Roskoski, 2019). In addition, reduced protein expression of p21 and p27 has been documented in breast, colon, and gastric tumors (Roskoski, 2019). Our present findings indicate that amlodipine could inhibit A549 cell proliferation by inducing cell cycle arrest *via* modulation of cyclin D1, p-Rb, p27, and p21.

Furthermore, we observed that amlodipine attenuated EGFR phosphorylation in A549 cells, and this finding is consistent with the report by Yoshida *et al.* who suggested that amlodipine suppresses the phosphorylation of EGFR in A431 cells (Yoshida et al., 2010). EGFR activation transduces multiple downstream pro-oncogenic signaling pathways, including the PI3K/Akt/mTOR and Ras/Raf/MEK/ERK pathways (Wee and Wang, 2017), which results in the progression of the G1/S cell cycle (Wee and Wang, 2017).

Subsequently, we noted that amlodipine could affect A549 cells by inhibiting the phosphorylation of PI3K/Akt pathway proteins such as PDK1, Akt (both Thr308 and Ser473), mTOR, p70 S6K, and GSK-3β. The PI3K/Akt/mTOR signaling pathway is one of the most frequently activated signal transduction pathways in human cancers (Martini et al., 2014; Alzahrani, 2019). PI3K phosphorylates phosphatidylinositol 4,5-bisphosphate (PIP2) and converts it to the secondary messenger, phosphatidylinositol 3,4,5-triphosphate (PIP3). PIP3 then recruits a subset of signaling proteins with pleckstrin homology domains, such as PDK1 and Akt, to the plasma membrane (Porta et al., 2014). PDK1 phosphorylates Akt in its activation loop at threonine 308 (Thr308), and full activation of Akt requires phosphorylation at serine 473 (Ser473) *via* mTORC2 activation (Martini et al., 2014; Alzahrani, 2019). Akt directly phosphorylates mTORC1 at Ser2448 (Navé et al., 1999), and activated mTORC1 regulates protein synthesis and cell survival by directly phosphorylating 4E-binding protein 1 and p70 S6K (Gibbons et al., 2009). Akt triggers a network that positively regulates G1/S cell cycle progression via GSK-3β inactivation, leading to increased cyclin D1 and decreased p27Kip1 (Liang and Slingerland, 2003). Dynamic regulated phosphorylation, which reduces the activity of GSK-3, occurs on Ser 9 of GSK-3β, and this N-terminal serine phosphorylation is mediated by Akt (Hermida et al., 2017).

Additionally, we observed that amlodipine effectively inhibited the phosphorylation of c-Raf and ERK, without impacting total ERK levels. The Ras/Raf/MEK/ERK pathway also plays an important role in cell proliferation and survival during several stages of cancer (Asati et al., 2016). Raf proteins are crucial components of the Ras/Raf/MEK/ERK signaling cascade and contain three isoforms; C-Raf (also called Raf-1), B-Raf, and A-Raf (Degirmenci et al., 2020). Activated C-Raf phosphorylates downstream MEK, which activates ERK by phosphorylating both Tyr and Thr regulatory sites (Vandamme et al., 2014; Muta et al., 2019; Guo et al., 2020). ERK1/2 are terminal kinases in MAPK signaling and are located in the cytoplasm when the signaling pathway is inactive. Once activated, ERK1/2 translocates to the nucleus and regulates the activity of several transcription factors, finally mediating cell growth, differentiation, and migration (Shin et al., 2018; Guo et al., 2020). In the present study, we found that amlodipine decreased A549 cell migration in a wound-healing assay. Thus, our results suggest that amlodipine suppresses A549 cell proliferation and migration by inhibiting EGFR-mediated PI3K/Akt and Raf/ERK pathways.

Several clinical studies have indicated that a single agent can present several limitations during the treatment of various cancers. Combination therapy involves the simultaneous administration of two or more therapeutic agents. Combination therapy has shown promising activity in cancer treatment; for example, sorafenib combined with other targeted agents, chemotherapy, and radiotherapy exhibits synergistic antitumor activity (Ibrahim et al., 2012), and gefitinib combined with carboplatin plus pemetrexed can improve progression-free survival when compared with gefitinib alone in patients with NSCLC (Hosomi et al., 2020). Our group has recently reported that combination therapy is beneficial in cancer treatment, such as biomimetic small-molecule self-assembly of the PI3K inhibitor, ZSTK474, integrated with the immunomodulator indomethacin to amplify anticancer efficacy (Zhang et al., 2022) and hydroxychloroquine combined with the PI3K inhibitor BKM120 to induce synergistic effects against tumor cells by manipulating reactive oxygen species clearance and homologous recombination repair processes independent of autophagy (Peng et al., 2021). Herein, we demonstrated that amlodipine acts synergistically with gefitinib by inducing cell cycle arrest in the G0/G1 phase of A549 cells. Compared with amlodipine or gefitinib treatment alone, combination therapy with amlodipine and gefitinib could significantly decrease p-Rb and increase p27 levels. In addition, amlodipine acts synergistically with gefitinib to downregulate the phosphorylation of Akt (Ser473), Akt (Thr308), and mTOR without impacting total Akt and mTOR levels, compared with either amlodipine or gefitinib alone, thus leading to the inhibition of cell cycle progression. Our results indicate that combined treatment with amlodipine and gefitinib exhibited a more potent antitumor effect than monotherapy suppressing A549 cell proliferation.

Furthermore, we investigated the tumor-inhibitory effect of amlodipine in combination with gefitinib in an *in vivo* mouse model. We found that combining amlodipine and gefitinib could enhance the antitumor effects without inducing evident toxicity. Tumors size was decreased, along with a reduction in Ki67-positive staining of the tumor tissue.

Collectively, these findings indicate that amlodipine is a distinct antitumor agent in combination with gefitinib against NSCLC proliferation both *in vivo* and *in vitro*. To the best of our knowledge, this is the first study to combine amlodipine with gefitinib. However, the precise molecular targets of amlodipine alone and in combination with gefitinib on anticancer effects in A549 cells remain elusive in the present study and will be investigated in our future work. Our findings support the potential utility of amlodipine treatment for NSCLC, and amlodipine in combination with gefitinib may be feasible for clinical use, potentially providing a new therapeutic strategy for NSCLC.

## Data Availability

The original contributions presented in the study are included in the article/Supplementary Material, further inquiries can be directed to the corresponding authors.
